# Fall risk and its associated factors among hypertensive older adults in Dhulikhel municipality, Nepal

**DOI:** 10.1007/s40520-025-03205-2

**Published:** 2025-10-30

**Authors:** Prabha Shrestha, Yunika Acharya, Indu Poudel, Lee Smith

**Affiliations:** 1 Department of Nursing, Kathmandu University School of Medical Sciences, Dhulikhel, Kavrepalanchowk Nepal; 2 Research and Development Division, Dhulikhel Hospital, Dhulikhel, Kavrepalanchowk, Nepal; 3 Center for Health Performance and Wellbeing, Anglia Ruskin University, Cambridge, UK; 4 Faculty of Medicine, Department of Public Health, Biruni University, Istanbul, Turkey

**Keywords:** Risk of fall, Hypertension, Older adults, Social support, Living arrangement

## Abstract

**Introduction:**

Falls are a leading cause of disability and mortality among older adults, particularly in Low- and Middle-Income Countries (LMICs) like Nepal. Despite the growing number of hypertensive older adults, evidence on their fall risk remains limited. This study assessed the prevalence and factors associated with fall risk among hypertensive older adults residing in Nepal.

**Method:**

A cross-sectional study was conducted among randomly selected hypertensive older adults (*n* = 186) in the Dhulikhel municipality, Nepal. Fall risk was assessed using the Timed Up and Go (TUG) test, with a cut-off of ≥ 14 s indicating high risk. Multivariable logistic regression was performed to identify associated factors.

**Result:**

Risk of fall was prevalent among 27% of older adults. After adjustment for potential confounders, with each year increase in age the risk of fall was 1.1 times higher (*p* < 0.01, 95% CI 1.05,1.2), for those with poor social support compared to those with good social support risk of fall was 4.1 times higher (*p* = 0.007, 95% CI 1.4, 11.7), for those who were not Living with family members compared to those who were Living with family members it was 0.1 times lower (*p* = 0.03, 95% CI 0.05, 0.6).

**Conclusion:**

Targeted fall prevention strategies should account for both physical risk factors like age and contextual elements such as living arrangements. Further research is needed to understand the protective mechanisms among older adults living alone in the LMICs.

## Introduction

Falls are one of the leading causes of morbidity, disability, and premature death among older adults globally, posing a significant public health challenge [1]. Each year, an estimated 684,000 people die from falls worldwide, with older adults accounting for the greatest number of fatal falls [1]. Over 80% of these deaths occur in Low- and Middle-Income Countries (LMICs) [1]. 

In Nepal, the aging population is increasing, and with it, age related health risks [2, 3]. The 2019 STEPS Survey showed that around 32.9% of adults aged 45 and above had hypertension, with prevalence increasing sharply with age [4]. Existing studies in Nepal have shown fall prevalence among older adults ranging from 8.2% to 52.4% [5, 6]. Emerging research suggests that every 10 mmHg increment in systolic blood pressure (SBP) is associated with poorer cognitive function, regardless of stroke or cardiovascular history [7]. This highlights the interconnected risks of hypertension, cognitive impairment, and falls in older age. Hypertension is a known risk factor for both vascular cognitive impairment and Alzheimer’s disease, the two most common causes of dementia [8]. This underscores the importance of early and sustained blood pressure control, not only for cardiovascular health but also for maintaining cognitive function and reducing fall risk in later life.

While several studies have explored the risk of falls among the general older adult population, limited attention has been given to hypertensive older adults, a group particularly vulnerable due to the interplay of aging and chronic illness. Despite the high burden of both falls and hypertension, access to comprehensive geriatric care and integrated fall prevention strategies remains scarce in Nepal. Understanding fall risk among hypertensive older adults is essential for developing targeted interventions and informed policies aimed at preventing falls and enhancing the quality of life in this population. Therefore, our study seeks to assess the prevalence of falls and identify associated risk factors among hypertensive older adults in Dhulikhel municipality, Nepal.

## Method

### Study design and settings

We conducted a cross-sectional study among hypertensive older adults aged 60 years and above from Dhulikhel municipality, Nepal. Dhulikhel Municipality is in the central part of Nepal, 30 km east of Kathmandu the capital city of Nepal. This includes 55 square kilometers of land with a total population of 34,858 people [9]. 

## Participants

We selected Dhulikhel Municipality using a convenience sampling method and simple random sampling to select the participants from the Healthy City Initiative study [10]. We randomly selected 186 older adults using Stata software from the Healthy City Initiative database of Dhulikhel Municipality. We included individuals aged 60 years and above, as the Government of Nepal defines older adults as those aged 60 or older [11]. We included only those with self reported hypertensive patients. We assessed cognitive function using the Rowland Universal Dementia Assessment Scale (RUDAS) and excluded individuals who showed signs of cognitive decline and who were bed bound [12]. 

To calculate the sample size, we considered that 61.2% of older adults in Nepal are at risk of falling [6]. Using a 95% confidence level and a 7% margin of error, we estimated the required sample size as 186, based on the formula:

n = (z² × p × q)/e² = (1.96² × 0.61 × 0.38)/0.07² = 186,

where z = 1.96, *p* = 0.61, q = 0.38, and e = 0.07.

We obtained written informed consent from all participants. Those who were unable to sign provided a thumbprint along with the caretaker’s signature. The Kathmandu University Institutional Review Committee (KU-IRC) approved this study (approval number 21/25).

## Data collection

We identified study respondents using data from the Healthy City database [10]. After obtaining the contact details of hypertensive patients who met the inclusion criteria, we reached out to them via phone and briefly explained the study objectives. Once they provided informed consent, we scheduled a mutually convenient time for data collection. Trained research assistants then conducted face-to-face interviews at the participants’ homes in Nepali language, between February and April 2025, using a structured questionnaire. We ensured that the timing of the interviews suited the participant’s availability and made efforts to create a relaxed and informal atmosphere to encourage open and comfortable discussions.

## Measures

Socio-demographic questions were adopted from a previously conducted national survey of Nepal [13]. 

We assessed BP readings using a Microlife automatic blood pressure monitor. Prior to measurement, participants were instructed to avoid caffeine, exercise, and smoking for at least 30 min and to empty their bladder. To ensure accuracy, both the participant and the observer remained silent during the rest and measurement periods. Participants were asked to remove any clothing covering the upper arm to allow proper cuff placement. Blood pressure was measured while the participant was seated comfortably in a chair with their back supported, feet flat on the floor, and back upright. Blood pressure was categorized as uncontrolled when the blood pressure readings for systolic and diastolic were above 140 mmHg or 90 mmHg, respectively [14]. 

We assessed social support using 8 items modified Medical Outcomes Study Social Support Survey (mMOSSS).The mMOSSS measures instrumental and emotional social support [15]. This Likert scale consists of 8 items ranging from 1 (none of the time) to 5 (all of the time) [15]. Total scores range from 1 to 40 and higher scores represent good social support [15]. The Nepali version of the mMOSSS was used herein [16]. The score was transformed into 100 [15]. A score more than 80 is considered good social support, 60–80 is considered fair social support and less than 60 is considered poor social support [17]. 

We assessed the risk of falls using the Timed Up and Go (TUG) test, a functional mobility assessment that evaluates how quickly an individual can stand up from a chair, walk a short distance, turn, return, and sit down again. For this test, participants sat in a standard armchair (with or without armrests), with a 3-meter (approximately 10 feet) distance marked on the floor in front of them [18]. On the command “Go,” participants stood up, walked at a comfortable and safe pace to the marker, turned around, walked back, and sat down with their back resting against the chair. We used a stopwatch to record the time taken in seconds [18]. A time of 14 s or more indicated a high risk of falls [18–20]. 

### Data analysis

Categorical data were reported in frequency and percentage; and numerical data with means and standard deviation. We used univariable and multivariable logistic regression models to assess the association between risk of falls with hypertension status, socio demographic, and level of social support. In the multivariable model, we adjusted the potential confounders based on a prior Literature review. We reported crude and adjusted odds ratios with a 95% confidence interval and p-value. All analyses were conducted using STATA version 14.0 (Stata Corp., College Station, Texas, USA) for cleaning, coding, and statistical analysis.

### Result

The mean age of the participants was 71.4 (6.7) years. More than half were female (58.6%) and married (66.6%). Over half of the participants (54.3%) were able to read and write, and more than 70% had attained at least a basic level of education. In terms of ethnicity, 51.1% identified as Janjati. The majority of participants (91.4%) followed the Hindu religion. Regarding Living arrangements, 75.2% were living with family members. (Table 1)

**Table 1:** Socio-demographic characteristics of the hypertensive older adults (*n* = 186)

Characteristics	n (%)
Age Mean (SD)	71.4 (6.7)
Sex	
Female	109 (58.6)
Male	77 (41.4)
Marital status	
Married	124 (66.6)
Not married	62 (33.4)
Education level	
Able to read and write	101 (54.3)
Not Able to read and write	85 (45.7)
Level of education	
Basic education	74 (73.2)
Secondary education and above	27 (26.8)
Ethnicity	
Janjati	95 (51.1)
Bhramin/chhetri	89 (47.8)
Dalit	2 (1.1)
Religion	
Hindu	170 (91.4)
Non Hindu	16 (8.6)
Living arrangement	
Living with family member	140 (75.2)
Not living with family member	46 (24.8)

N frequency % percentage

**Fig. 1:**
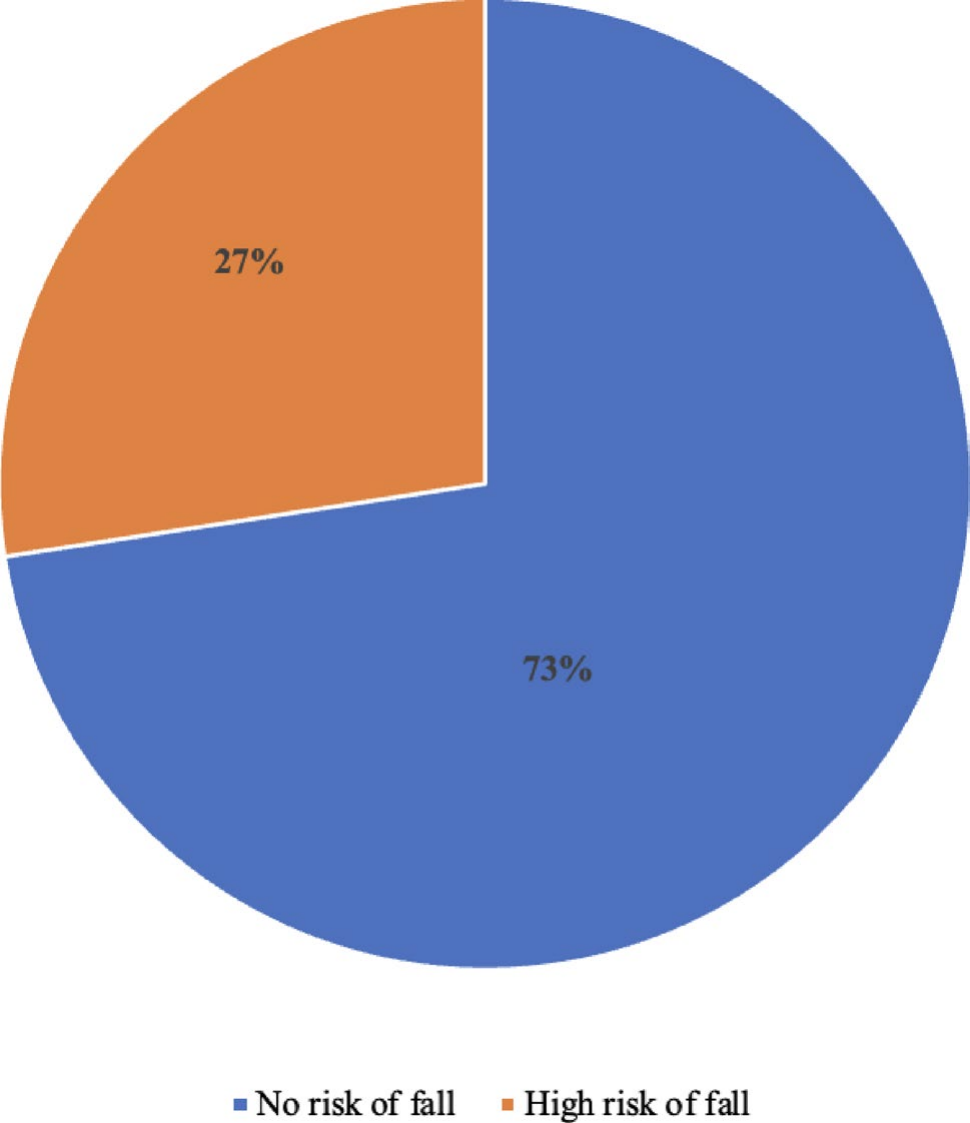
Risk of fall among hypertensive older adults (*n* = 186)

The risk of fall among participants was 27% (Fig. 1). The mean TUG score was 12.03 (4.7) seconds.

More than half of the participants had uncontrolled blood pressure (60.2%) and good social support (67.2%) (Table 2).

**Table 2:** Blood pressure status and levels of social support among hypertensive older adults (*n* = 186)

Characteristics	*n* (%)
Hypertension status	
Controlled blood pressure	74 (39.8)
Uncontrolled blood pressure	112 (60.2)
Level of social support	
Good social support	125 (67.2)
Fair social support	31 (16.7)
Poor social support	30 (16.1)

N frequency % percentage

After adjusting for potential confounders, with each year increase in age the risk of fall was higher by 1.1 times (*p* < 0.01, 95% CI 1.05,1.2) and lowered by 0.1 times among those who were not living with family members compared to those who were living with family members (*p* = 0.006, 95% CI 0.05, 0.6). Similarly, the odds of risk of fall was higher by 4.1 times among those with poor social support compared to those with good social support after adjustment for potential confounders (*p* = 0.007, 95% CI 1.4, 11.7) (Table 3).

**Table 3:** Factors associated with risk of fall among hypertensive older adults (*n* = 186)

	Bivariable analysis			Multivariable analysis		
Characteristics	OR	95% CI	P value	OR	95% CI	P value
Age	1.1	1.06,1.1	**< 0.01**	1.1	1.05, 1.2	**< 0.01**
Sex						
Female	Ref			Ref		
Male	0.5	0.2,1.09	0.09	0.3	0.1,1.04	0.06
Marital status						
Never married	Ref			Ref		
Married	0.5	0.2,1.08	0.08	1.3	0.5,3.03	0.5
Education						
Able to read and write	Ref			Ref		
Not able to read and write	2.07	1.07,3.9	**0.02**	0.7	0.2,1.9	0.5
Religion						
Hindu	Ref			Ref		
Non-Hindu	1.6	0.5,4.8	0.3	1.2	0.3,4.5	0.6
Living arrangement						
With family members	Ref			Ref		
Not with family members	0.2	0.09, 0.6	**0.006**	0.1	0.05,0.6	**0.006**
Hypertension status						
Controlled	Ref			Ref		
Uncontrolled	1.1	0.5, 2.2	0.6	1.06	0.4,2.2	0.8
Social support						
Good social support	Ref			Ref		
Fair social support	1.1	0.4,2.8	0.7	1.6	0.5,4.4	0.3
Poor social support	2.8	1.2,6.6	**0.01**	4.1	1.4,11.7	**0.007**

## Discussion

In this study, a little over one fourth of older adults were at risk of falls. The risk of fall was higher among those with increasing age and those with poor social support and lower among those who are not living with family members.

In our study, the prevalence of fall risk among older adults was found to be 27%, indicating that over one-fourth of the study population may be vulnerable to falling. This prevalence is relatively low compared to findings from similar studies conducted in other regions of Nepal where, a study among older adults in Bharatpur, Chitwan, reported that 59.2% were at high risk of falls, suggesting a considerably greater burden in that setting [5]. Similarly, in Birtamod, Jhapa, 61.2% of elderly individuals aged 70–79 years were classified as high risk using the TUG test [6]. The possible explanation for this might be due to the variation in study populations as our study focuses on hypertensive older adults aged 60 years and above whereas other studies focused on general elder population with varying health conditions and age group. Such differences in samples may have resulted in lower fall risk in our study.In comparison to international data, our findings are somewhat higher than those reported in large-scale studies from neighboring countries. The Longitudinal Aging Study of India (LASI), which analyzed data from 28,710 elderly individuals, found a prevalence of fall risk at 11.43% [21]. However, community-based studies in different regions of India have reported higher fall risk prevalence rates, ranging from 26% to 37%, which align more closely with our findings [22]. Similar to the present study, in Bangladesh, a study conducted among the rural elderly population found a fall prevalence of 30% among individuals aged 65 years and older [23]. The variation in fall risk prevalence across these studies can be explained by differences in several factors. Population characteristics such as health status, and presence of chronic conditions differ across settings and influence fall risk levels. Additionally, the methods used to assess fall risk vary across studies. For example, self-reported measures may differ compared to objective functional tests like the TUG. Environmental and cultural factors such as living conditions, community infrastructure, and social support systems and disparities in access to healthcare and availability of fall prevention programs may contribute to differences observed across populations.

In this study with each year increase in age the risk of fall was higher, similar to other studies conducted in Nepal, India and published systematic reviews [6, 22, 24]. As people age, there is a natural decrease in muscle strength, motor function, balance, joint flexibility, and sensory abilities such as vision and hearing, resulting changes impair mobility and stability, increasing fall risk [25]. While prior studies have indicated that living alone increases the risk of falls among older adults, our findings showed a lower fall risk among individuals not living with family members [26, 27]. This discrepancy may be influenced by cultural and contextual factors. In Nepal, increasing out-migration of youth and family members has led many older adults to live independently. However, those who continue to live alone may be relatively healthier due to being more functionally active. It is possible that older adults living without family may engage in more routine physical activity as they manage daily tasks independently, contributing to better mobility and lower fall risk. In contrast, those living with family members may receive more assistance with daily activities, potentially resulting in reduced physical activity levels and diminished functional mobility over time. Poor social support was associated with risk of falls in our study. This finding aligns with longitudinal analyses showing that social isolation significantly predicts falls even after adjusting for demographic factors [28]. Social support may help prevent falls by providing emotional encouragement, assistance with daily tasks, and promoting physical activity, which in turn improves balance and mobility [29]. Similarly, a cross-sectional study conducted in Brazil in 2024 found that elderly individuals with higher perceived social support particularly in the affective and positive social interaction dimensions had a significantly reduced risk of Falls, with fall risk reductions ranging from 4% to 28% depending on the type of social support received [30]. 

Our study provides important insights into the factors associated with fall risk among hypertensive older adults in Nepal. By employing a structured and objective tool to assess fall risk, we were able to examine how individual characteristics such as age, living arrangements, and social support influence vulnerability to falls. The multivariable analysis showed independent association between risk of falls and various exposures, even after adjusting for potential confounders. However, this study has certain limitations. First, the sample was restricted to hypertensive older adults from the Healthy city Initiative, which may not represent the experiences of healthier individuals with other disease conditions. Secondly, blood pressure was measured only once for each participant due to logistical and resource constraints; to minimize measurement error, standardized procedures were followed during measurement, and trained personnel conducted all readings. Thirdly, although various variables were included, due to resource constraints, this study did not account for other potentially important factors such as environmental hazards, physical activity levels, medication use, or nutritional status. Despite these limitations, the use of an objective assessment tool and analysis across multiple variables enhances the relevance of the findings for informing fall prevention strategies in similar settings.

## Conclusion

This study found that more than one-fourth of older adults with hypertension are at risk of falls. The risk was higher among individuals of advanced age, those with poor social support and lower among those not living with family members. These findings emphasize the need for targeted interventions that not only address physical risk factors but also consider social support systems. Designing fall prevention programs that focus on older adults particularly those with poor social support may be crucial in reducing fall related incidents and enhancing overall wellbeing.

## Data Availability

Data are available upon reasonable request.
